# Role of Generative Artificial Intelligence in Personalized Medicine: A Systematic Review

**DOI:** 10.7759/cureus.82310

**Published:** 2025-04-15

**Authors:** Aashish Mishra, Anirban Majumder, Dheeraj Kommineni, Chrishanti Anna Joseph, Tanay Chowdhury, Sathish Krishna Anumula

**Affiliations:** 1 Computer Science and Information Technology, Eastern Kentucky University, Richmond, USA; 2 Research, Amazon Science Education, Scottsdale, USA; 3 Systems Analytics, Hanker Systems, Chantilly, USA; 4 Anesthesiology and Perioperative Medicine, University of Pittsburgh, Pittsburgh, USA; 5 Data Science, Amazon Web Sciences (AWS) Generative AI Innovation Center, Sammamish, USA; 6 Research and Development, IBM Corporation, Detroit, USA

**Keywords:** generative artificial intelligence, healthcare technology, machine learning in medicine, personalized medicine, precision health

## Abstract

Precision medicine presents challenges in data collection, cost, and privacy as it tailors treatments to each patient's unique genetic and clinical profile. With its ability to produce realistic and confidential patient data, generative artificial intelligence (AI) offers a promising avenue that could revolutionize patient-centric healthcare. This systematic review aims to assess the role of generative AI in personalized medicine. Following the Preferred Reporting Items for Systematic Reviews and Meta-Analyses (PRISMA) guidelines, we searched PubMed, Web of Science, Scopus, CINAHL, and Google Scholar, identifying 549 studies. After removing duplicates and applying eligibility criteria, 27 studies were found relevant and were included in this systematic review. Generative adversarial networks (GANs) were the most commonly used models (16 studies), followed by variational autoencoders (VAEs; seven studies). These models were primarily applied to drug response prediction, treatment effect estimation, biomarker discovery, and patient stratification. Generative AI models have shown significant promise in revolutionizing personalized medicine by enabling precise treatment predictions and patient-specific therapeutic insights. Despite their potential, challenges related to model validation, interpretability, and bias remain. Future research should prioritize large-scale validation studies using diverse datasets to enhance the clinical applicability and reliability of these AI-driven approaches.

## Introduction and background

Precision medicine differs from conventional clinical treatment by focusing on tailoring therapeutic approaches to each patient’s unique genetic and clinical characteristics [1]. This method integrates clinical data with genetic profiling to develop personalized diagnostic and treatment strategies, aligning clinical decisions with an individual’s medical history and current health status [2].

Generative artificial intelligence (AI) is a type of AI focused on creating new data, content, or solutions using various formats, including text, graphics, and man-made data [3]. Machine learning (ML) models, with a focus on deep learning approaches, are used in this cutting-edge sector to generate outputs that are both original and realistic [4]. These results are obtained by identifying and reproducing structures and patterns in the currently available data. Generative AI, especially through sophisticated deep Galerkin methods (DGMs) like variational autoencoders (VAEs) and generative adversarial networks (GANs), has emerged as a crucial tool in precision medicine [5]. These advanced AI models skillfully address challenging problems like privacy concerns, data shortages, and difficulties in modeling intricate human health data. These models greatly improve the interpretation and analysis of data by producing synthetic data on patients that preserve authenticity and realism, thus promoting precision medicine [6].

Despite its potential, the integration of generative AI into personalized medicine raises several challenges. Issues related to model transparency, data bias, interpretability, regulatory compliance, and ethical considerations remain major concerns. The reliability of AI-driven predictions heavily depends on the quality and diversity of training datasets, as biases in data collection can lead to skewed or misleading outcomes [7]. Additionally, the black-box nature of some generative AI models poses challenges in clinical adoption, as healthcare professionals require clear justifications for AI-driven decisions. Addressing these concerns is crucial to ensure the safe, effective, and equitable deployment of generative AI in clinical practice [8].

Given the growing interest in generative AI and its applications in personalized medicine, a systematic review is essential to synthesize existing evidence, evaluate methodological advancements, and identify potential gaps in research. This systematic review aims to comprehensively assess the role of generative AI in personalized medicine, examining its impact on diagnosis, treatment, and patient outcomes. Additionally, the review will critically evaluate the reliability, generalizability, and ethical considerations of AI-driven models to provide insights into their future implications in healthcare. By systematically analyzing the current literature, this review will contribute to the ongoing discourse on AI integration in precision medicine and inform future research directions and policy frameworks.

## Review

Methodology

Study Design

This systematic review was conducted following the Preferred Reporting Items for Systematic Reviews and Meta-Analyses (PRISMA) [9] guidelines to ensure methodological rigor and transparency. A structured approach was used to identify, evaluate, and synthesize existing literature on the role of generative AI in personalized medicine.

Eligibility Criteria

The inclusion criteria were established based on the Population, Intervention, Comparison, Outcomes, and Study Design (PICOS) framework. Studies were included if they focused on patients or healthcare settings where generative AI was applied to personalized medicine, encompassing areas such as genomics, drug discovery, disease diagnosis, and treatment optimization. The intervention of interest was the use of generative AI models such as GANs, VAEs, and transformer-based architectures for healthcare applications. Studies comparing generative AI with conventional statistical methods, traditional ML approaches, or clinical decision-making without AI intervention were included. The primary outcomes considered were predictive accuracy, improvements in patient outcomes, efficiency in drug discovery, and ethical considerations related to AI implementation.

This review included randomized controlled trials (RCTs), observational studies, retrospective analyses, cohort studies, and computational modeling studies. Studies that were non-peer-reviewed, such as preprints, editorials, and commentaries, were excluded. Only studies published in English were considered, and those lacking methodological transparency or complete data were omitted.

Search Strategy

A comprehensive literature search was conducted across multiple electronic databases, including PubMed, Web of Science, Scopus, CINAHL, and Google Scholar. A combination of Medical Subject Headings (MeSH) terms and free-text keywords was used to refine the search. The primary search terms included “Generative AI”, “Generative Adversarial Networks”, “Transformer models”, “AI in Precision Medicine”, “AI-driven Drug Discovery”, “AI in Genomics”, “Personalized Treatment AI”, and “Machine Learning in Healthcare”. Boolean operators (AND, OR, NOT) were applied to optimize the search strategy. Additionally, the reference lists of included studies were manually reviewed to identify any relevant studies not captured in the initial search.

Study Selection

All retrieved studies were imported into EndNote reference management software (Clarivate, Philadelphia, PA, USA), and duplicates were removed. The screening process was carried out in two phases. In the first phase, titles and abstracts were reviewed by two independent researchers to identify potentially relevant studies. The second phase involved a full-text review, where studies meeting the eligibility criteria were assessed in detail. Any disagreements between the reviewers were resolved through discussion or consultation with a third reviewer. The entire selection process was documented using a PRISMA flow diagram to provide transparency on study inclusion and exclusion.

Data Extraction

A standardized data extraction form was used to systematically collect relevant information from the included studies. Extracted data included study details, study design, sample size, type of generative AI model, application domain, comparison methods, outcome measures, and key findings. Additionally, ethical considerations related to bias, data privacy, and regulatory compliance were recorded. The data extraction process was independently conducted by two reviewers to ensure accuracy, and discrepancies were resolved through discussion.

Risk of Bias Assessment

The risk of bias for the included studies was assessed using the Prediction Model Risk of Bias Assessment Tool (PROBAST). This tool evaluates the risk of bias across four domains: participants, predictors, outcome, and analysis. Each study was independently reviewed by two researchers, and any discrepancies were resolved through discussion. Studies were categorized as having low, moderate, or high risk of bias based on the presence of limitations in one or more domains. Particular attention was given to the analysis domain, as inappropriate statistical methods or overfitting could significantly impact the validity of findings. The final risk of bias assessments were summarized in a tabular format for transparency.

Data Synthesis and Analysis

Due to variations in study designs and outcome measures, a narrative synthesis approach was adopted. Findings were organized thematically based on key areas, including types of generative AI models used, their applications in disease diagnosis and prognosis, their role in drug discovery and personalized treatment, and the ethical and regulatory challenges associated with their implementation.

Ethical Considerations

Since this systematic review did not involve direct human or animal research, formal ethical approval was not required. However, ethical concerns associated with the use of AI in personalized medicine, including data privacy, algorithmic bias, interpretability, and regulatory compliance, were critically analyzed. Ensuring transparency and fairness in generative AI applications remains a key priority for its integration into personalized healthcare.

Results

Search Results

**Figure 1:**
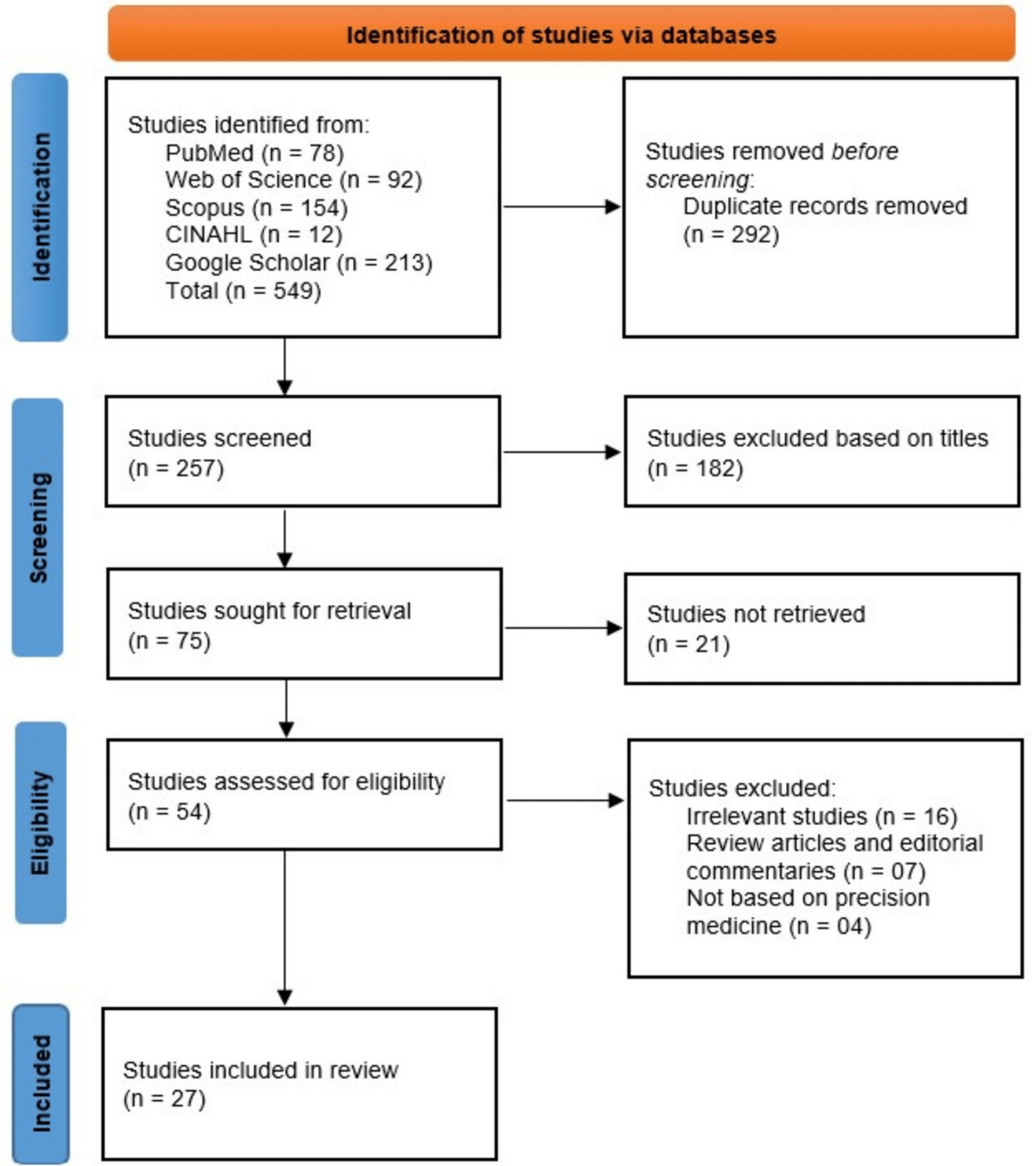
PRISMA flowchart of the studies identified from different databases PRISMA: Preferred Reporting Items for Systematic Reviews and Meta-Analyses.

The initial database search identified 549 records from PubMed (n = 78), Web of Science (n = 92), Scopus (n = 154), CINAHL (n = 12), and Google Scholar (n = 213). After removing 292 duplicate records, 257 studies were screened based on titles, of which 182 were excluded for irrelevance. The remaining 75 studies were sought for retrieval, with 21 unavailable, leaving 54 for full-text eligibility assessment. Among these, 16 were excluded as irrelevant, seven were review articles or commentaries, and four did not focus on precision medicine. Ultimately, 27 studies met the inclusion criteria and were included in the systematic review (Figure 1).

Characteristics of Included Studies

The included studies employed a range of generative models, with GANs being the most prevalent (16 studies), followed by VAEs (seven studies). Other models included U-Net, latent Dirichlet allocation (LDA), and large language models (LLMs), each represented in one or two studies. The majority of the studies adopted retrospective computational modeling designs, while a smaller number utilized deep learning-based approaches, predictive modeling, or benchmarking frameworks (Table 1).

**Table 1:** Characteristics of included studies ^VAE: variational autoencoder, Dr. VAE: drug response variational autoencoder, GANITE: generative adversarial nets for individualized treatment effect, MCGAN: Monte Carlo generative adversarial network, S-VQ-VAE: structured vector quantized variational autoencoder, GAN: generative adversarial network, GP-GAN: growth prediction generative adversarial network, ADS-GAN: anonymization through data synthesis using GAN, WGAN: Wasserstein generative adversarial network, CVAE-GAN: conditional variational autoencoder generative adversarial network, GANDA: generative adversarial network for distribution of nanoparticles, ECG: electrocardiogram, omicsGAN: omics data-integrated generative adversarial network, U-Net: U-shaped convolutional neural network, U-HPNet: U-Net-based high-performance network, LDA: latent Dirichlet allocation, MixEHR: mixed-membership model for electronic health records, MS-ASGAN: multi-scale attention-based synthetic generative adversarial network, CSAM-GAN: cross-scale attention mechanism GAN, MOICVAE: multi-omics integration conditional variational autoencoder, DRAGONET: drug gene interaction network generator, Wasserstein-GA: Wasserstein generative adversarial architecture, BrainStatTrans-GAN: brain statistical transformation GAN, AttentionGAN: attention-based generative adversarial network, GANCMLAE: GAN with convolutional multi-level autoencoder, AD: Alzheimer’s disease, MCI: mild cognitive impairment, CCGAN: clinical completion generative adversarial network, GAN-boosted SSL: GAN-boosted semi-supervised learning, SCAN: small-data cancer network, CGAN: conditional generative adversarial network, SCGAN: sparse conditional generative adversarial network, GluGAN: glucose generative adversarial network, LLMs: large language models, ChatGPT: chat generative pretrained transformer, BioMedLM: biomedical language model, SSL: semi-supervised learning, EHR: electronic health record.^

Author	Publishing year	Study design	Underlying generative model	Applied generative model	Focused application
Rampášek et al. [10]	2019	Retrospective computational modeling study	VAE	Dr. VAE	Personalized drug response prediction
Ge et al. [11]	2020	Retrospective computational study with simulation-based validation	GANITE	MCGAN	Personalized treatment effect (ITE)
Xue et al. [12]	2020	Computational modeling study in systems biology	VAE	VAE, S-VQ-VAE	Representation of cellular states from gene expression data
Elazab et al. [13]	2020	Computational imaging study	GAN	GP-GAN	Growth prediction of gliomas (brain tumors)
Yoon et al. [14]	2020	Computational study	GAN	ADS-GAN	Anonymization through data synthesis
Barbiero et al. [15]	2021	Computational modeling study	GAN	WGAN	Production realistic gene expression samples
Sui et al. [16]	2021	Deep learning-based radiogenomic study	VAE and GAN	CVAE-GAN	Analyze the correlation between lung cancer imaging and gene expression data
Zhang et al. [17]	2021	Computational modeling study	GAN	GANDA	Generation of intratumoral nanoparticles distribution (nps)
Piacentino et al. [18]	2021	Computational modeling study	GAN	GAN-based ECG	Anonymize private healthcare data
Ahmed et al. [19]	2021	Computational modeling study	GAN	omicsGAN	Improved disease phenotype prediction
Rafael-Palou et al. [20]	2022	Predictive modeling study	U-Net	U-HPNet	Predicting the progression of lung nodules
Ahuja et al. [21]	2022	Unsupervised machine learning in clinical informatics	LDA	MixEHR	Large-scale automatic phenotyping using electronic health record (EHR) data
Jahanyar et al. [22]	2023	Computational biology study	GAN	MS-ASGAN	Evaluating tabular biomedical data generated by GANs
Shi et al. [23]	2023	Computational predictive modeling study	GAN	CSAM-GAN	Predicting prognostic outcomes in cancer using multimodal data
Wang et al. [24]	2023	Computational predictive modeling study	VAE	MOICVAE	Predict cancer drug response
Yamanaka et al. [25]	2023	Computational predictive modeling study	VAE	DRAGONET	Generate new drug candidate molecules
Strack et al. [26]	2023	Computational predictive modeling study	GAN	Wasserstein-GA	Monitor brain tumor changes
Gao et al. [27]	2023	Computational predictive modeling study	GAN	BrainStatTrans-GAN	Generate corresponding healthy images of patients, further used to decode individualized brain atrophy
Moon et al. [28]	2023	Retrospective in silico study	GAN	AttentionGAN	Predict short-term anatomical treatment outcomes for different anti-vascular endothelial growth factor agents
Shi et al. [29]	2023	Retrospective computational imaging study	GAN	GANCMLAE	Precisely detect individual brain atrophy patterns in Alzheimer’s disease (AD) and mild cognitive impairment (MCI)
Bernardini et al. [30]	2023	Retrospective observational study	GAN	CCGAN	Clinical data imputation
Li et al. [31]	2023	Retrospective observational study	GAN	GAN-boosted SSL	Improve prediction models trained on electronic health records (EHRs)
Hsu and Lin [32]	2023	Retrospective computational study	VAE	SCAN	Predicting cancer patient prognosis using small medical datasets
Zhou et al. [33]	2024	Retrospective computational modeling study	CGAN	SCGAN	Counterfactual explanations in breast cancer prediction
Zhu et al. [34]	2023	Retrospective computational modeling study	GAN	GluGAN	Personalized glucose monitoring
Benary et al. [35]	2023	Diagnostic study	LLMs	ChatGPT, Galactica, Perplexity, and BioMedLM	Supporting tool in precision oncology
Huang et al. [36]	2023	Retrospective benchmarking study	LLMs	ChatGPT-3 and ChatGPT-4	Benchmarking ChatGPT-4 on a radiation oncology in-training exam and Red Journal Gray Zone cases

The applications of generative AI in these studies were highly diverse, reflecting the broad potential of these technologies in personalized medicine. Several studies focused on drug response prediction, leveraging models such as drug response variational autoencoder (Dr. VAE) [10] and multi-omics integration conditional variational autoencoder (MOICVAE) [24] to tailor treatments based on individual patient data. Another prominent area was treatment effect estimation, where models like generative adversarial nets for individualized treatment effect (GANITE) [11] and sparse conditional generative adversarial network (SCGAN) [33] provided insights into individualized therapeutic outcomes. Medical imaging synthesis and analysis were also well-represented, with studies like growth prediction generative adversarial network (GP-GAN) [13] and generative adversarial network for distribution of nanoparticles (GANDA) [17] demonstrating the utility of generative AI in modeling tumor growth and nanoparticle distribution. Additionally, biomarker and gene expression modeling were explored using VAEs and GANs to decode cellular states [12] and generate synthetic gene expression data [15].

Clinical data imputation and phenotyping emerged as another critical application, with studies such as clinical completion generative adversarial network (CCGAN) [30] and mixed-membership model for electronic health records (MixEHR) [21] addressing challenges in electronic health record (EHR) analysis. Disease progression and prognosis prediction were also key themes, with models like U-Net-based high-performance network (U-HPNet) [20] and cross-scale attention mechanism GAN (CSAM-GAN) [23] offering tools for forecasting outcomes in conditions such as lung nodules and cancer. Finally, two studies highlighted the emerging role of LLMs, including ChatGPT, in clinical decision support, particularly in precision oncology [35,36]. These studies underscored the ability of generative AI to handle small datasets [32], integrate multimodal data [23], and facilitate personalized treatment planning.

Overall, the included studies showcased the transformative potential of generative AI across various facets of personalized medicine. The predominance of GANs and VAEs reflected their versatility in tasks ranging from data synthesis to predictive modeling [10,12-20,22-24,26-31,33,34], while the inclusion of LLMs pointed to new frontiers in AI-assisted healthcare [35,36]. The findings collectively emphasized the growing importance of generative AI in addressing complex, individualized medical challenges.

Risk of Bias Assessment

The PROBAST assessment revealed variation in the risk of bias among the included studies. Of the 27 studies, 6 were classified as low risk, 12 as moderate risk, and 9 as high risk of bias. Common sources of bias included poor model transparency, insufficient validation, and limited generalizability due to reliance on small or non-diverse datasets. The analysis domain showed the highest prevalence of bias, particularly in studies that did not account for overfitting or lacked external validation. Notably, studies with low risk of bias had well-documented methodologies, rigorous validation strategies, and comprehensive reporting, enhancing their reliability in the context of personalized medicine applications (Table 2).

**Table 2:** Risk of bias assessment using the PROBAST tool

Study	Participants	Predictors	Outcome	Analysis	Overall risk of bias
Rampášek et al. [10]	Low	Moderate	Moderate	Low	Moderate
Ge et al. [11]	Moderate	High	Moderate	Moderate	Moderate
Xue et al. [12]	Low	Moderate	Low	Low	Moderate
Elazab et al. [13]	High	Low	Moderate	High	High
Yoon et al. [14]	Moderate	Moderate	Moderate	Moderate	Moderate
Barbiero et al. [15]	Low	Low	Moderate	Low	Low
Sui et al. [16]	Moderate	Moderate	Moderate	High	Moderate
Zhang et al. [17]	High	Moderate	High	High	High
Piacentino et al. [18]	Low	Low	Low	Low	Low
Ahmed et al. [19]	Moderate	Moderate	High	Moderate	High
Rafael-Palou et al. [20]	High	High	High	High	High
Ahuja et al. [21]	Low	Moderate	Moderate	Low	Moderate
Jahanyar et al. [22]	Moderate	Low	Moderate	High	Moderate
Shi et al. [23]	High	High	Moderate	High	High
Wang et al. [24]	Low	Moderate	Low	Moderate	Moderate
Yamanaka et al. [25]	Moderate	Moderate	Moderate	Moderate	Moderate
Strack et al. [26]	High	High	High	High	High
Gao et al. [27]	Low	Low	Low	Low	Low
Moon et al. [28]	Moderate	Moderate	Moderate	High	Moderate
Shi et al. [29]	High	High	Moderate	High	High
Bernardini et al. [30]	Low	Moderate	Low	Moderate	Moderate
Li et al. [31]	Moderate	Moderate	Moderate	Moderate	Moderate
Hsu and Lin [32]	High	High	High	High	High
Zhou et al. [33]	Low	Low	Low	Low	Low
Zhu et al. [34]	Moderate	Moderate	Moderate	Moderate	Moderate
Benary et al. [35]	High	High	Moderate	High	High
Huang et al. [36]	Low	Moderate	Low	Moderate	Moderate

Discussion

The systematic review highlights the rapid evolution and diverse applications of generative AI in personalized medicine. By synthesizing findings across domains such as drug response prediction, medical imaging synthesis, clinical data imputation, and prognostic modeling, this review underscores the versatility of generative models in addressing individualized healthcare challenges. Below, we interpret these results through five thematic lenses, comparing them with broader trends in AI-driven medical research and emphasizing their contributions to advancing precision medicine. 

Foundations of Generative AI in Precision Medicine

GANs and VAEs dominated the included studies, collectively accounting for 23 out of 27 studies. GANs were particularly prevalent, reflecting their unparalleled ability to synthesize high-dimensional data, such as medical images (e.g., gliomas in GP-GAN) [13] and gene expression profiles (e.g., omicsGAN) [19]. VAEs, used in seven studies, excelled in probabilistic modeling and dimensionality reduction, enabling tasks like drug response prediction (Dr. VAE) [10] and cellular state representation (S-VQ-VAE) [12]. 

The dominance of GANs aligns with their historical success in image synthesis, but their adaptation to non-image data, such as tabular EHRs (CCGAN) [30] or glucose time series (GluGAN) [34], demonstrates their expanding utility. Comparatively, VAEs’ strength in handling uncertainty and latent space interpretability makes them ideal for scenarios requiring probabilistic outputs, such as treatment effect estimation (SCGAN) [33]. These findings mirror broader trends in AI research, where GANs and VAEs are often juxtaposed: GANs prioritize data fidelity, while VAEs emphasize structured latent representations. 

Existing studies outside this review, such as those by Goodfellow et al. [37] on GANs and Kingma and Welling [38] on VAEs, established these models as cornerstones of generative AI. However, the included works extend their applications beyond foundational tasks. For instance, GANs’ role in anonymizing data (anonymization through data synthesis using GAN (ADS-GAN)) [14] and VAEs’ use in small dataset learning (small-data cancer network (SCAN)) [32] highlight innovations tailored to healthcare’s unique constraints, such as privacy concerns and data scarcity. 

Generative AI as a Multimodal Integrator

A striking theme across the studies is the integration of multimodal data to address complex medical questions. For example, Sui et al. [16] fused radiogenomic data using a conditional variational autoencoder generative adversarial network (CVAE-GAN) architecture to correlate lung cancer imaging with gene expression, while Shi et al. [23] employed attention-based GANs to combine genomic, imaging, and clinical data for cancer prognosis. These approaches reflect a paradigm shift from single-modality analyses to holistic, patient-specific modeling. 

The emphasis on multimodal integration resonates with broader AI research, where models like transformers have revolutionized natural language processing (NLP) and vision tasks. However, generative models offer unique advantages in healthcare: they can impute missing data (MixEHR) [21], synthesize complementary datasets (e.g., healthy brain images in brain statistical transformation GAN (BrainStatTrans-GAN)) [27], and simulate counterfactual scenarios (SCGAN) [24]. Such capabilities address critical gaps in traditional methods, which often struggle with incomplete or siloed data. 

Compared to non-generative multimodal frameworks (e.g., convolutional neural networks (CNNs) for imaging combined with logistic regression for clinical data), generative models provide a unified architecture for joint learning. For instance, U-HPNet by Rafael-Palou et al. [20] used a U-Net-based hierarchical probabilistic network to model lung nodule progression, leveraging both imaging and temporal data. This contrasts with conventional predictive models that treat modalities separately, often leading to fragmented insights. 

Generative AI in Low-Resource Settings

A significant subset of studies addressed the challenge of limited data, a pervasive issue in healthcare. VAEs like MOICVAE [24] and SCAN [32] demonstrated robust performance in predicting drug responses and cancer prognosis using small datasets, while GANs such as MS-ASGAN [22] generated synthetic gene expression data to augment schizophrenia research. These approaches mitigate reliance on large-scale datasets, which are often impractical in niche medical domains. 

This focus on data efficiency contrasts with conventional deep learning, which typically requires millions of samples. For example, traditional CNNs for tumor detection demand extensive labeled imaging data, whereas GP-GAN [13] achieved accurate glioma growth predictions using longitudinal MRIs from limited patients. Similarly, LLMs like ChatGPT [35] provided decision support in precision oncology without requiring task-specific training data, instead leveraging pretrained knowledge. 

Existing studies in federated learning and transfer learning have also tackled data scarcity, but generative models offer a distinct advantage: they create new data rather than merely adapting existing models. For instance, synthetic ECG generation via GANs [18] preserves patient privacy while enabling algorithm training, a solution unmatched by non-generative methods. 

Tailoring Interventions With Generative AI** **

The reviewed studies exemplify how generative AI enables granular personalization. For example, GANITE [11] estimated individualized treatment effects (ITEs) by simulating counterfactual outcomes, while attention-based generative adversarial network (AttentionGAN) [28] predicted anatomical changes in macular degeneration patients based on anti-vascular endothelial growth factor** **(VEGF) agent specifics. These models move beyond population-level insights to deliver patient-specific predictions, a cornerstone of precision medicine. 

This marks a departure from classical statistical models, such as Cox regression for survival analysis, which identify average risk factors but fail to account for individual heterogeneity. In contrast, CSAM-GAN [23] incorporated multimodal data to predict cancer outcomes at the patient level, capturing nuances like genetic mutations and treatment history. Similarly, drug gene interaction network generator (DRAGONET) [25] designed de novo drug molecules tailored to individual gene expression profiles, a feat unattainable with traditional QSAR (quantitative structure-activity relationship) models. 

Comparisons with non-generative personalized approaches, such as reinforcement learning for treatment optimization, reveal trade-offs. While RL excels in dynamic decision-making, generative models provide a probabilistic framework for exploring hypothetical scenarios (e.g., “What if Patient X received Drug Y?”). This is exemplified by the counterfactual explanations in breast cancer prediction of SCGAN [33], offering clinicians actionable insights into alternative therapeutic paths. 

Expanding the Frontiers of Clinical Decision Support

Though limited to two studies, the inclusion of LLMs like ChatGPT [35] and biomedical language model (BioMedLM) [36] signals an emerging frontier. These models demonstrated proficiency in interpreting clinical literature, answering oncology exams, and providing gray-zone case analyses, suggesting their potential as real-time decision aids. 

The ability of LLMs to parse unstructured text, such as EHR notes or research articles, complements traditional generative models focused on structured data. For instance, while GANs synthesize images and VAEs model drug responses, LLMs contextualize findings within the broader medical knowledge base. Huang et al. [36] illustrated this by benchmarking ChatGPT-4 on radiation oncology exams, achieving performance comparable to human trainees. This capability aligns with efforts like BioBERT [39] but extends beyond information retrieval to generative reasoning. 

However, LLMs’ reliance on pretrained knowledge raises questions about hallucination and reproducibility, a challenge noted in Benary et al. [35]. Unlike GANs/VAEs, which are trained on domain-specific data, LLMs may generate plausible but unverified recommendations. This contrasts with models like Dr. VAE [10], whose outputs are grounded in perturbation experiments, highlighting a need for hybrid frameworks that combine LLMs’ linguistic prowess with generative models’ data-driven rigor. 

Synthesis and Comparative Reflections** **

The reviewed studies collectively underscore generative AI’s capacity to address three pillars of personalized medicine: prediction (e.g., prognosis), prescription (e.g., drug design), and personalization (e.g., counterfactual analysis). Compared to non-generative AI, these models excel in scenarios requiring synthetic data generation, multimodal integration, and individualized output, capabilities critical for precision medicine’s evolution. 

Notably, the proliferation of GANs and VAEs mirrors their established roles in general AI research but adapts them to healthcare’s ethical and technical constraints (e.g., privacy-preserving synthetic data). Meanwhile, the nascent adoption of LLMs opens avenues for democratizing expert-level knowledge, albeit with caveats around reliability. 

When juxtaposed with existing systematic reviews on AI in healthcare, such as those focusing on diagnostic imaging or predictive analytics, this review highlights generative models’ unique value in creating rather than merely analyzing data. For instance, while prior studies celebrated CNNs for tumor detection, generative models like GP-GAN [13] and GANDA [17] advance the field by simulating disease progression and therapeutic responses, offering dynamic insights static models cannot provide. 

## Conclusions

GANs and VAEs emerged as foundational tools, excelling in tasks ranging from synthetic data generation (e.g., anonymized ECGs, tumor growth simulations) to probabilistic modeling of drug responses and cellular states, while their adaptability to multimodal data integration bridged gaps between imaging, genomics, and clinical records. The ability of these models to address data scarcity, through synthetic data augmentation, small-dataset learning, and privacy-preserving synthesis, demonstrates their critical role in overcoming resource limitations in healthcare. Furthermore, the shift toward patient-specific interventions, exemplified by counterfactual treatment effect estimation and de novo drug design, highlights generative AI’s unparalleled capacity to tailor insights at the individual level. The nascent integration of LLMs, though limited in scope, signals a paradigm shift toward AI-driven clinical decision support, blending linguistic reasoning with data-driven precision. Collectively, these studies illustrate how generative AI redefines personalized medicine by transforming raw data into actionable, individualized insights, thereby enhancing prediction, prescription, and patient-centric care. This synthesis not only validates the versatility of existing models but also sets the stage for a future where generative technologies become indispensable in addressing medicine’s most complex, individualized challenges.
